# Effects of High-Fructose Diets on Central Appetite Signaling and Cognitive Function

**DOI:** 10.3389/fnut.2015.00005

**Published:** 2015-03-04

**Authors:** Katrien Lowette, Lina Roosen, Jan Tack, Pieter Vanden Berghe

**Affiliations:** ^1^Laboratory for Enteric Neuroscience, Department of Clinical and Experimental Medicine, Translational Research Center for Gastrointestinal Disorders (TARGID), Leuven, Belgium; ^2^Department of Clinical and Experimental Medicine, Translational Research Center for Gastrointestinal Disorders (TARGID), Leuven, Belgium

**Keywords:** fructose, appetite, cognition, HFCS, memory

## Abstract

The consumption of fructose has increased tremendously over the last five decades, which is to a large extent due to the development of high-fructose corn syrup (HFCS), a commercial sugar additive that contains high amounts of free fructose. HFCS is often added to processed food and beverages partly because it is a powerful sweetener but even more so because the production is cheap. Although fructose in combination with fiber, vitamins, and minerals, as present in fruits, is a healthy source of energy, isolated fructose, in processed food products has been associated with several health disorders such as insulin resistance and hypertension. Apart from its metabolic consequences, a growing body of literature suggests that free fructose can also affect neuronal systems. High-fructose intake may on the one hand affect central appetite regulation by altering specific components of the endocannabinoid system. On the other hand, it appears to impact on cognitive function by affecting phosphorylation levels of insulin receptor, synapsin 1, and synaptophysin. The present report reviews the recent evidence showing a negative effect of free fructose consumption on central appetite control, as well as cognitive function.

## Introduction

Fifty years ago, it was already recognized that too much sugar can be harmful for human health: “pure, white, and deadly,” is how John Yudkin (1) described this macronutrient after he showed the association between sugar consumption and coronary heart diseases. Today, the leading sugar in terms of negative health effects is fructose (2). This monosaccharide, also known as fruit sugar, is twice as sweet as glucose and used to be consumed in a balanced fructose-to-glucose ratio, together with the fiber, vitamins, and minerals as present in fruits. However, increasing amounts of free fructose are used in Western diets, with some soft drinks even containing twice as much fructose compared to glucose (3). The addition of fructose to all kinds of food dates back to 1957, when a commercial method to convert glucose in fructose was developed. This made the production of high-fructose corn syrup (HFCS), containing both glucose and fructose as monosaccharides, relatively easy (4). The relatively low production cost of HFCS has facilitated the ever increasing intake of fructose via processed food and beverages.

Along with increased fructose consumption, also the prevalence of metabolic diseases such as obesity and diabetes type 2 has steadily risen. High-fructose diets were recognized to cause oxidative stress, decreased glucose tolerance, insulin resistance, and hypertension (2, 5, 6). Moreover, elevations in fructose consumption have been shown to impair the signaling of appetite hormones and neuronal health (7). Therefore, the present review summarizes the recent findings concerning fructose-induced effects on central appetite signaling and cognitive functions.

## Fructose Facts

Fructose or fruit sugar is a monosaccharide with the same molecular formula as glucose (C_6_H_12_O_6_). However, they differ structurally, because glucose contains an aldehyde while fructose a ketone group. Because of its structure, fructose appears more frequently in the active open-chain configuration, which results in a greater reactivity with amines as described in the Maillard reaction (8). This increased reactivity finally leads to more advanced glycation end products (AGEs), linked to diabetic complications and neurodegeneration (9, 10).

**Figure 1:**
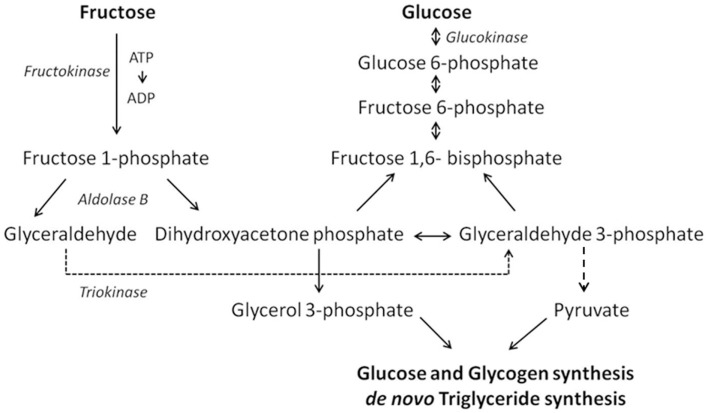
**Schematic of the crucial steps in the hepatic fructose and glucose metabolism**.

Due to these structural differences, the metabolism of glucose and fructose is quite different. More specifically, fructose is preferentially metabolized in the liver while glucose is consumed primarily in the brain. After ingestion, both glucose and fructose are transported from the intestinal lumen to the blood by members of the glucose transport (GLUT) family. This GLUT family consists of 14 members of which seven are able to transport fructose, namely GLUT5, GLUT2, GLUT7, GLUT9a/b, GLUT8, GLUT11, and GLUT12. Among these transporters, GLUT5 is the only one with specificity for fructose, and is expressed in the intestine, liver, kidney, testis, skeletal muscle, adipose tissue, and brain. Intestinal GLUT5 mediates the transport of fructose across the apical membrane while GLUT2 is responsible for basolateral transportation. Once across the epithelium, fructose enters the bloodstream and is transported to the liver via the portal vein (4). Because glucokinase has a higher affinity for glucose, phosphorylation of fructose to fructose 6-phosphate is inhibited by glucose (11). Therefore, almost all fructose is metabolized in the liver via the fructose 1-phosphate pathway involving the enzymes fructokinase, aldolase, and triokinase (Figure 1). The intermediates of this pathway, dihydroxyacetone phosphate and glyceraldehyde 3-phosphate, can react to become glucose 6-phosphate, which in turn can be either metabolized into glucose to fuel cellular processes or into glycogen to be stored in the liver. However, when liver glycogen is replenished, the fructose intermediates are directed toward *de novo* triglyceride synthesis by converting dihydroxyacetone phosphate and glyceraldehyde 3-phosphate to glycerol 3-phosphate and pyruvate, respectively (12). Thus, metabolically the consequences of a diet rich in added fructose (e.g., as HFCS) are analogous to those of a fat-rich diet. This is the reason why fructose has been identified as a player in metabolic syndrome, insulin resistance, high blood pressure, and adverse neuronal effects, the latter of which will be discussed in the next sections (13).

## Fructose and Appetite

Initially, fructose was proposed to be a safe sugar for diabetic patients because fructose metabolism was considered to be independent of insulin secretion, and therefore, fructose intake would only induce a limited rise in glucose levels (12). However, recent studies of Lindqvist et al. (7) showed that serum insulin levels of fructose-drinking (23% solution) rats increased significantly after 2 weeks. Apart from insulin, a 2-week period of high-fructose consumption did also modulate other appetite-regulating peptides such as the appetite-inducing hormone ghrelin and the appetite-inhibiting hormone leptin. Although the increase in leptin levels could be mimicked by glucose consumption (23% solution), the increase in ghrelin levels was exclusive to fructose intake. Interestingly, both sugars, in a 23% solution, induced a significant increase in caloric intake and body weight after 2 weeks (7); while consumption of a 15% fructose solution for 6 weeks, also in rats, did not affect body weight, suggesting a concentration and/or duration-dependent mechanism (14). Additional research, including different sugar concentrations for varying diet durations, is needed to elucidate the link between appetite hormone levels, feeding behavior, and body weight.

Apart from modulating peripherally released appetite hormones, elevated sugar levels also affect hypothalamic appetite peptides (7). Peptide YY (PYY), neuropeptide Y (NPY), and pro-opiomelanocortin (POMC) mRNA expression was shown to be significantly decreased after 2 weeks consumption of a 23% fructose or glucose solution; whereas cannabinoid 1 (CB1) receptor mRNA expression was significantly upregulated in response to fructose only (7). Also other components of the endocannabinoid signaling pathway are affected by sugar intake. Erlanson-Albertsson and Lindqvist (15) found that consumption of 23% sugar solutions (sucrose, glucose, and fructose) affects the levels of hypothalamic endocannabinoids by altering the expression of synthesizing and degrading enzymes. More specifically, after 1 week a decrease in phospholipase C (PLC) and an increase in monoglyceride lipase (MGLL) mRNA expression for each of the sugars were observed; while only fructose induced an increase in fatty acid amide hydrolase (FAAH) and diacylglycerol lipase (DAG) 1β, and a decrease in DAG1α mRNA. Although the link between these molecular changes and appetite behavior needs further research, these results suggest that fructose consumption, apart from affecting appetite, may also help determining what type of nutrients is consumed, as the endocannabinoid system is crucial in the rewarding aspect of food intake (15). However, whether and to what extent food choices are modulated by fructose consumption is at the moment still speculative and more research is needed to fully clarify this possibility. Moreover, caution needs to be taken when interpreting changes in mRNA levels only, as Rojo et al. (16) showed no changes in CB1 receptor functionality after intake of palatable foods. Apart from the reward system, fructose consumption also modulates the serotonergic system, which is an important contributor to psychological wellbeing (17). More specifically, consumption of a 30% fructose diet for 8 weeks resulted in a decrease in serotonin reuptake transporter (SERT) protein levels in mouse duodenum. Interestingly, in depression research, SERT-deficient mice are used as a relevant model for depression, suggesting a link between fructose consumption and psychological effects (18). Whether fructose restriction may be associated with an improved psychological state needs to be determined.

Fructose-induced modulation of appetite signaling peptides was not only observed in rodent models but also in humans. Page et al. (19) found that one single fructose drink (25% solution) induced an increase in PYY levels, without change in insulin as was the case with glucose (25%). Neither single drinks of glucose nor fructose induced detectable differences in plasma leptin and ghrelin levels. In addition, using magnetic resonance imaging (MRI), they found that, contrary to fructose, only glucose was able to quickly (within 15 min) mediate satiety by reducing brain activity in specific appetite-regulating regions (e.g., hypothalamus) (19). Interestingly, the glucose drink induced an increase in functional connectivity between hypothalamus and striatum suggesting that glucose improves the communication between appetite control centers. In contrast, the fructose-drink only stimulated an increase in connectivity between hypothalamus and thalamus, which is thought to be insufficient to induce satiety (19).

## Fructose and Cognition

The effects of fructose on cognitive function remain somewhat unclear. While older studies suggest a protective effect similar to glucose (20), the recent increase in fructose consumption has led to a rise in specific studies addressing nutrient-related changes in memory and cognition, revealing an association between fructose consumption and cognitive impairment.

Rodent studies have shown that fructose intake can lead to brain insulin resistance, which leads to diminished cognitive function. More specifically, consumption of fructose during 6 weeks reduced phosphorylation levels of the insulin receptor (IR), leading to impaired insulin signaling in hamsters (60% fructose solid food) and rats (15% fructose liquid) (14, 21). Brain insulin resistance is associated with memory impairment in rats as suggested by the increased latency times in the Barnes maze test (14). Additional evidence to corroborate the harmful effect of fructose on cognitive function was provided by showing diminished phosphorylation of cAMP-response element binding (CREB) and synapsin I and reduced synaptophysin levels after a 6 weeks consumption of 15% fructose solution. Of note, they demonstrated that all fructose-induced cognitive impairments were ameliorated by adding omega-3 fatty acid to the diet (14). Although it is not clear what the direct link is, if any, between omega-3 and the fructose metabolism, it is noteworthy that intake of known beneficial food components can counteract, albeit in an indirect way – the adverse effects of high-fructose intake. This, in its own right, is of high value with respect to improving dietary advice. Ross et al. (13) confirmed the fructose-induced cognitive impairment in male rats, using a spatial water maze test. They showed that the animals, after a solid 60% fructose diet, needed more time to reach the target, performed fewer target approaches, and spent less time in the target quadrant. Together, these observations direct to the conclusion that excess fructose consumption leads to impaired spatial memory in male hamsters and rats. There are, however, also some contradicting data, cognitive testing using an operant bar-pressing task in fructose-fed (15%, 3 months) mice showed improved memory and learning processes (22); suggesting that apart from concentration-dependent differences, there might also be a species-dependent effect. Moreover, also sex may be important, as Bruggeman et al. (23) found that female animals were protected against fructose-induced cognitive impairments, as they did not perform any worse in the spatial water maze test after a 60% fructose diet (up to 144 days) (23). This sex difference of fructose-induced disorders has also been shown by Vasudevan et al. (24) in the context of metabolic disorders. They suggested that estrogen may counteract the effects of fructose (60% solid, 7 weeks), since treatment of male rats with estrogen improved insulin sensitivity and reduced body mass (24). However, the protection in female animals could also be independent from fructose since estrogen has also been shown to be directly neuroprotective as it could reverse streptozotocin-induced cognitive impairments (25).

Apart from its effects on learning and memory, fructose can also affect neurogenesis as shown in the hippocampus as well as in the nucleus tractus solitarius (NTS) (26, 27). Using 5′-bromo-2′-deoxyuridine (BrdU), van der Borght et al. (26) evaluated the amount of newborn neurons in different dietary conditions. They found that 4 weeks of fructose and sucrose consumption (23% solutions) significantly reduced hippocampal neurogenesis, while glucose consumption did not have a reducing effect (26). Next, Rafati et al. (27) demonstrated that neuronal loss after fructose consumption (10% solution for 6 weeks) was observed in the NTS. Additional research is needed to understand whether the fructose-induced reduction in neurogenesis is associated with altered appetite control and/or cognitive impairment.

## Conclusion

**Figure 2:**
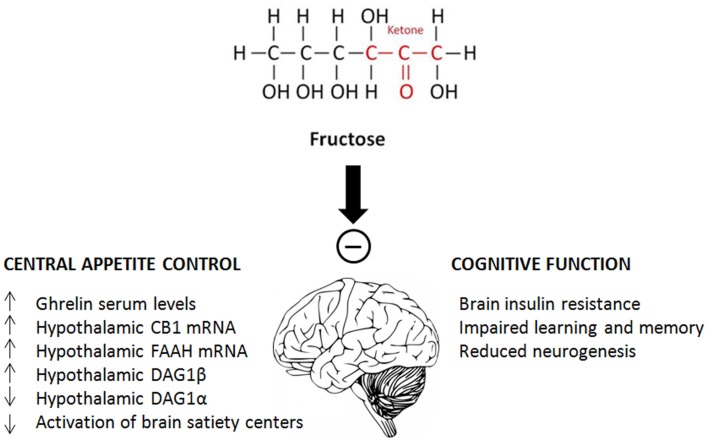
**Schematic summary of the main effects of fructose on central appetite control and cognitive function**.

Although not all studies come to exactly the same conclusion, sufficient evidence has accumulated over the last 10 years to indicate that fructose, in certain concentrations and mainly in males has a significant impact on brain and cognitive functions. On the one hand, fructose intake affects appetite control by increasing ghrelin serum levels and hypothalamic CB1 mRNA, and decreasing the activation of brain satiety centers. On the other hand, it leads to brain insulin resistance, impaired learning and memory, and reduced neurogenesis (Figure 2). Despite some contradictory results, care needs to be taken with respect to the intake of processed foods and beverages since recent tests showed that the free fructose content in popular soft drinks is still increasing, with some beverages containing up to 50% more fructose than glucose (3). In order to fully understand the effects that these high concentrations of fructose exert on central and peripheral neuronal pathways, and to be able to better link molecular and behavioral fructose-induced changes, more specific studies using relevant fructose concentrations are required. Improved understanding of the effects of fructose consumption is crucial to improve dietary advice with respect to the intake of purified sugars.

## Conflict of Interest Statement

The authors declare that the research was conducted in the absence of any commercial or financial relationships that could be construed as a potential conflict of interest.
